# Single-cell profiling reveals immune disturbances landscape and HLA-F-mediated immune tolerance at the maternal-fetal interface in preeclampsia

**DOI:** 10.3389/fimmu.2023.1234577

**Published:** 2023-10-03

**Authors:** Fangyuan Luo, Fulin Liu, Yingzhe Guo, Wenming Xu, Yilin Li, Jun Yi, Thierry Fournier, Séverine Degrelle, Hedia Zitouni, Isabelle Hernandez, Xinghui Liu, Yu Huang, Jun Yue

**Affiliations:** ^1^ Department of Obstetrics and Gynecology, Sichuan Academy of Medical Sciences & Sichuan Provincial People’s Hospital, University of Electronic Science and Technology, Chengdu, China; ^2^ Department of Obstetrics and Gynecology, West China Second University Hospital of Sichuan University, Chengdu, China; ^3^ Department of Obstetrics/Gynecology, Key Laboratory of Obstetric, Gynecologic and Pediatric Diseases and Birth Defects of Ministry of Education, West China Second University Hospital of Sichuan University, Chengdu, China; ^4^ Sichuan Provincial Key Laboratory for Human Disease Gene Study, Center for Medical Genetics, Department of Laboratory Medicine, Sichuan Academy of Medical Sciences & Sichuan Provincial People’s Hospital, University of Electronic Science and Technology, Chengdu, China; ^5^ School of Medical and Life Sciences, Chengdu University of Traditional Chinese Medicine, Chengdu, China; ^6^ Department of Obstetrics and Gynecology Nursing, Sichuan Academy of Medical Sciences & Sichuan Provincial People’s Hospital, University of Electronic Science and Technology, Chengdu, China; ^7^ Pathophysiology & Pharmacotoxicology of the Human Placenta, Pre & Postnatal Microbiota, Université Paris Cité, Paris, France; ^8^ Inovarion, Paris, France; ^9^ Laboratory of Human Genome and Multi-factorial Diseases, Faculty of Pharmacy of Monastir, Monastir, Tunisia

**Keywords:** preeclampsia, placental decidua, single-cell RNA sequencing, immune tolerance, maternal-fetal interface, human leucocyte antigen

## Abstract

**Background:**

Preeclampsia is a pregnancy-specific disorder that always causes maternal and fetal serious adverse outcome. Disturbances in maternal immune tolerance to embryo at the maternal-fetal interface (MFI) may be associated with preeclampsia onset. Recent studies have revealed the reduced expression pattern of HLA-F at the MFI in preeclampsia, while the mechanism of it mediating maternal fetal immune tolerance has not been revealed.

**Methods:**

Single-cell RNA sequencing on placental decidua was performed to reveal the immune disturbances landscape at the MFI in preeclampsia. Human Jar cells and NK-92MI cells were employed to study the role of HLA-F in trophoblasts and lymphocyte.

**Results:**

A total of 101,250 cells were classified into 22 cell clusters. Disease-related IGFBP1+SPP1+ extracellular villus trophoblast (EVT) was identified in the preeclamptic placental decidua, accompanied by newly discovered immune cellular dysfunction such as reduced ribosomal functions of NK populations and abnormal expression of antigen-presenting molecules in most cell clusters. Certain genes that are characteristic of the intermediate stage of myeloid or EVT cell differentiation were found to have unexplored but important functions in the pathogenesis of preeclampsia; specifically, we detected enhanced cell cross-talk between IGFBP1+SPP1+ EVT2 or SPP1+M1 cells and their receptor cell populations at the MFI of PE patients compared to controls. With respect to HLA-F, mIF staining confirmed its reduced expression in PE samples compared to controls. Over-expression of HLA-F in Jar cells promoted cell proliferation, invasion, and migration while under-expression had the opposite effect. In NK-92MI cells, over-expression of HLA-F increased the secretion of immunoregulation cytokines such as CSF1 and CCL22, and promoted adaptive NKG2C+NK cell transformation.

**Conclusions:**

We revealed the immune disturbance landscape at the MFI in preeclampsia. Our findings regarding cellular heterogeneity and immune cellular dysfunction, as revealed by scRNA-seq, and the function of HLA-F in cells provide new perspectives for further investigation of their roles in the pathogenesis of preeclampsia, and then provide potential new therapeutic target.

## Introduction

Preeclampsia (PE) affects approximately 2–4% of pregnant women worldwide, resulting in approximately 46,000 maternal deaths and 500,000 fetal and neonatal deaths annually (1). PE is a disease with multiple etiologies, including maternal intestinal disease, viral infection, obesity, autoimmune system disease, and disturbances in immune tolerance at the maternal-fetal interface (MFI), among others (2). The establishment and maintenance of a steady state of immune tolerance at the MFI is essential for a successful pregnancy. Beyond their role in PE, disturbances in maternal tolerance have also been found to contribute to other pregnancy complications such as spontaneous abortion during the first trimester and fetal growth restriction (3). The most important period for the establishment of immune tolerance is in the first trimester when the uterine spiral artery is remodeled during the process of placentation. At this time, extravillous trophoblasts (EVTs) of fetal origin actively interact with maternal–immune cells and vascular endothelial cells (4, 5). The main mechanisms of maternal-fetal immune tolerance during this period include (i) intense immune regulation and immunosuppression of specific immune cell populations in the decidua and (ii) the generation and release of anti-inflammatory and immunosuppressive hormones, cytokines, and immunomodulatory molecules by trophoblasts and immune cells.

Specifically, decidual stromal cells (DSC) regulate the microenvironment of the MFI during the first trimester by secreting a variety of cytokines, including vascular endothelial growth factor a (VEGFa) (6). Decidual CD16^-^CD56^+^ natural killer cells (dNK) and decidual CD16^-^CD163^+^ M2 macrophages (dMΦ) act synergistically to maintain maternal tolerance to embryos; for example, colony-stimulating factor 1/2 (CSF1/2) secreted by the former can stimulate the proliferation, differentiation, phagocytosis, and chemotactic activity of the latter, which in turn increase their interactions with other immune cells and modulate local and systemic inflammatory responses at the MFI (3). The only immune cells found in the placental villi are placental macrophages with a CD14^+^CD16^-^CD163^+^SPP1^+^ M2-like phenotype (Hofbauer cells, HBs), which play an important role in promoting and regulating placental blood vessel formation and growth, removing debris and harmful molecules that enter the placenta, and preventing transplacental infection (7). Decidual regulatory T cells (Treg) also act to maintain immune tolerance at the MFI and the balance of the decidual immune microenvironment (8). All of these circulating immune cells come into direct contact with the outer layer of the placental villi, the syncytial trophoblast (SCT), which is the main site for the synthesis and secretion of steroids and peptide hormones (9, 10). In the interaction between trophoblasts and immune cells, SCT acts as an immune barrier because it does not express major histocompatibility complex (MHC) class I and class II molecules and so is not recognized by CD8^+^ T cells (11). To prevent maternal immune rejection, the EVT expresses the human leukocyte antigens (HLA) HLA-G, HLA-E, HLA-F, and a small amount of HLA-C (but not antigenic HLA-A and HLA-B) (12). The interactions between these immunosuppressive molecules and CD4^+^ T cells lead to increased numbers of Treg cells, thereby promoting maternal immune tolerance (13). At the same time, HLA-G expressed by EVTs can induce, via inhibitory receptors, the secretion of a large number of growth-promoting factors by dNK cells, with benefits for fetal growth and development (14). Overall, these various findings represent considerable progress in our understanding of the complex mechanisms underlying maternal-fetal tolerance. However, the extent to which disturbances in the above immune regulatory mechanisms play a role in the pathogenesis of PE remains only incompletely understood.

Another important role of HLA molecules is in the interaction between EVTs and immune cells. The nonclassical HLA class I molecules—HLA-E, HLA-F, and HLA-G can present peptides and nonprotein antigens to immune cells, including T lymphocytes, nonclassical T cells, and NK cells (15–17). Among EVT-expressed HLA molecules, HLA-C and HLA-G have received more attention, while the functions of HLA-E and HLA-F in pregnancy and PE have only been poorly described (18). HLA-F is highly expressed in inflammatory-activated lymphocytes, and its open conformer has functions related to antigen presentation and immune activation; it can also activate NK cells to kill target cells infected by viruses through KIR3DS1 receptor (19–21). The HLA-F gene is located immediately downstream of, and shares some expression quantitative trait loci (eQTLs) with, the HLA-G gene, with which it is known to synergistically facilitate immune tolerance at the MFI (16). Previous studies have also shown that polymorphisms in the HLA-F gene can modify the expression of HLA-F at the MFI, thereby affecting the pregnancy rate in assisted reproduction and the incidence of spontaneous abortion (22–24). Therefore, we believe that HLA-F may play an underappreciated role in the pathogenesis of PE.

In the current study, single-cell RNA sequencing (scRNA-seq) was used to compare the cellular heterogeneity of placenta decidua obtained from PE patients and women with normal late pregnancy. The role of HLA-F in the establishment of maternal-fetal immune tolerance and its possible involvement in the pathogenesis of PE were also investigated. Our data reveal that the MFI of PE patients is characterized by inflammatory macrophages, dysfunctional NK cells, and EVTs associated with inflammatory fibrosis. This work also demonstrates that HLA-F facilitates immune tolerance through promoting EVT cell function and increasing the secretion of immunoregulatory cytokines by NK cells. Our findings could contribute to a better understanding of PE pathogenesis and inform the selection of potential therapeutic targets for future exploration.

## Materials and methods

### Ethics approval

The protocol presented here was reviewed and approved by the Ethics Committee of the Sichuan Provincial People’s Hospital (No.2022-311). Each patient provided her written informed consent regarding the sample’s experiment-related applications.

### Patients and specimens

PE was determined following the diagnostic criteria of Magee et al. (1). For scRNA-seq, last-trimester placental decidua was collected from two women with PE and two women with normal pregnancy in Sichuan Provincial People’s Hospital (Chengdu, China). Tissue was taken from the placental decidua within 5 min of the placenta being removed from the maternal uterus during cesarean section. In each case, four to five sites were sampled from the maternal surface of the placenta. A surgical blade was used to remove sections of decidual tissue that were approximately 1 mm thick and 0.5x1 cm^2^ in area; as much as possible, we avoided sampling the placental parenchyma. The validation cohort for analysis of HLA-F expression included 10 PE patients and 11 control patients with normal pregnancies. Samples were obtained following the procedure described above.

### scRNA-seq analysis

Samples of decidual tissue were used to create single-cell suspensions as described in (25), and the cell viability rate was determined to be above 90%. Library construction was performed using a BD Rhapsody™ Single-Cell Analysis System following the standard protocol provided by the manufacturer (BD Biosciences) (26). RNA sequencing was performed on an Illumina HiSeq 4000 machine and raw counts were aligned to human reference data (GRCh38 version). We used the BD Rhapsody WTA Analysis Pipeline (Version 1.10) to generate a filtered expression matrix for each sample, which was then analyzed and processed with the R package Seurat (version 4.0.3). All Seurat objects were subjected to the same quality filtering process, also conducted in Seurat. Cells with < 200 or > 7,500 detected genes were excluded from the analysis. The fraction of unique mitochondrial transcripts was less than 25%. Doublets in the cells were detected using the R package Doublet Finder with default parameters.

### Cell cluster analysis

The SCTtransform and glmGamPoi methods were used to remove batch effects between samples. Principal component analysis (PCA) of gene expression was used to reduce dimensionality preliminarily, and PCA results were further dimensionality reduced and visualized using the RunTSNE and RunUMAP functions of Seurat. The number of cell clusters was calculated for each case at resolutions of 0.1~1. Clustertree was used to show the number and trend of clusters at each resolution value, in order to help us select an appropriate resolution value for cell clustering. When viewed in combination with the cell population distributions shown by tSNE and UMAP, the results of resolution 0.5 were determined to be the most appropriate, and a total of 22 clusters were obtained. We used the FindAllMarkers function of Seurat to identify differentially expressed genes (DEGs) in each cluster (*P* value < 0.05). We then used the R package SingleR, combined with Maker annotation and reference (5), to infer cell type manually.

### Monocle trajectory analysis

Monocle 2 was used to infer the developmental trajectories of myeloid cells and EVT cells. All target cell clusters were separated from the Seurat object, transferred into SingleCellExperiment format, and then submitted for trajectory analysis with Monocle following the official tutorial (http://cole-trapnell-lab.github.io/monocle-release/docs/#constructing-single-cell-trajectories). Cell clusters 0, 16, 5, 10, and 21 were subjected to myeloid cell developmental trajectory analysis and clusters 1 and 4 underwent EVT cell developmental trajectory analysis.

### Cell-cell communication analysis

For the analysis of cell-cell communication, we used CellChat (1.1.0; http://www.cellchat.org/), a public repository of ligands, receptors, cofactors, and their interactions. For the cell-interaction analysis, expression levels were calculated relative to the total number of reads mapping to the same set of coding genes in all transcriptomes. The expression values were averaged within each single-cell cluster/cell sample.

### Multiplex immunofluorescence staining and quantitative analysis

Multiplex immunofluorescence (mIF) of placental decidual tissue was performed using a Four-color Fluorescence kit (Hunan AiFang Biotechnology Co., Ltd.)- based on the tyramide signal amplification (TSA) technology according to the manufacturer’s instruction. The following antibodies and dilutions were used: anti-HLA-F (Abcam #ab126624, 1:100), anti-HLA-G (Thermo Fisher #PA5-98143, 1:100), anti-HLA-G (Proteintech #66447-1-ig, 1:3000), anti-SPP1 (Abcam #ab214050, 1:2000), anti-ITGA5 (Abcam #ab150361, 1:1500), anti-CD14 (Abcam #ab221678, 1:1200), anti-CD44 (Abcam #254530, 1:2000), anti-IGF2 (AiFang #AF301007, 1:50), anti-IGFBP1 (Abcam #ab228741,1:250), anti-PSG3 (Abcam #ab154700, 1:200), and anti-COL4A2 (AiFang #03156, 1:200).

Decidual tissue from three patients with preeclampsia were used for mIF to investigate the ligand-receptor signaling exchange at MFI, and another 10 cases and 11 control samples were used for double immunofluorescence stain (dIF) to verify the expression of HLA-F and HLA-G. To quantify the dIF results, three images of each sample were captured in randomly selected fields of each IF section at magnification ×20 and used for the analysis of positive staining. The ImageJ image analysis system (National Institutes of Health, USA) was used to measure the integrated density (IntDen) and area of all the images collected and calculate the mean gray value (Mean) of each image. The average fluorescence intensity of the three images was calculated to obtain the average fluorescence intensity of each sample.

### Cells

The Jar cell line, a model of human placental villus cancer, and the human NK-92MI cell line were commercially obtained from Procell (Wuhan, China). Jar cells were cultured in DMEM containing 10% FBS (SH30084.03, HyClone Fetal Bovine Serum, Characterized, Australian origin; HyClone) and 1% double antibiotic (100 mg/L streptomycin and 100 U/mL penicillin) at 37°C in a 5% CO_2_ environment. Both the human Jar and NK-92MI cell lines had been authenticated using STR profiling within the previous three years. All experiments were performed with mycoplasma-free cells.

### Cell transfection

Jar cells and NK-92MI cells were transfected with an HLA-F pcDNA3.1-3xFlag plasmid (Flag-HLA-F) (RBO-ZL, XiangWu Bio Co., Ltd, Chengdu, China) or a control plasmid (Flag-NC) using Lipo2000 transfection reagent (Invitrogen, USA) according to the manufacturer’s instructions. The amount of plasmid transfection in a single well (6-well plate) was 2.5 µg and the concentration of siRNA was 50 nM. After 48 h, the cells were harvested and used for subsequent experiments. The HLA-F siRNA target gene sequences were as follows: 1) CAGAGGAGTTCAGGACCTA; 2) ACGTAGACGACACGCAATT; 3) TCACCCAGCGCTTCTATGA (Guangzhou Ribobio Co., Ltd, Guangzhou, China). Jar cells were transfected with either siRNA that targeted HLA-F (siRNA-HLA-F) or a nonspecific scrambled RNA sequence (siRNA-NC) using riboFECTTMCP Reagent (Guangzhou Ribobio Co., Ltd, Guangzhou, China) according to the manufacturer’s protocol, and analyzed three days later. The sequences of siRNA-NC were 5’-GGCUCUAGAAAAGCCUAUGCdTdT-3’ (Guangzhou Ribobio Co., Ltd, Guangzhou, China).

### Cell proliferation assay

A CCK8 assay was employed to test the viability of Jar cells transfected with Flag-HLA-F/Flag-NC or siRNA-HLA-F/siRNA-NC. In brief, approximately 5000 Jar cells were seeded into 96-well plates and cultured for 48 h. After reagent treatment, 10 µl of CCK-8 solution was added to each well, and cells were incubated for 2 h. Absorbance at 450 nm was measured using a microplate reader (BioTek^®^ ELx808IU, USA).

### Transwell assay

Jar cells were transfected with Flag-HLA-F/Flag-NC or siRNA-HLA-F/siRNA-NC, and then 2 ×10^4^ cells suspended in 200 µl of serum-free RPMI 1640 medium were seeded in the upper chamber of a transwell plate (Corning, USA, #3422). RPMI 1640 medium with 10% FBS was placed in the bottom chamber as the chemoattractant. For the transwell invasion assay, the transwell chamber was covered with 80 µl Matrigel mix (0.867 mg/ml) (BD Biosciences, USA). After being incubated for 16 h (migration assay) or 24 h (invasion assay), the upper chambers were fixed and stained using crystal violet (Kaigen, China) for 30 min. The cell lines were then photographed and counted in three fields. Each experiment was repeated three times and had three replicates.

### ELISA

HLA-F protein expression was evaluated in transfected NK-92MI cells (containing either an HLA-F over-expression plasmid or control plasmid) using a Human HLA-F ELISA kit (ZCi Bio, Shanghai, China). The cell supernatant of NK-92MI cells was also tested using the same kit. In these same cells, we also analyzed the protein expression of the cytokines CCL5, CSF-1, IFN-γ, TGG-β1, VEGF-a, and CCL22 using an ELISA kit (ZCi Bio, Shanghai, China) following the manufacturer’s instructions. In brief, the NK-92MI cells and their medium (supernatant) were harvested and used for measuring the concentration of target proteins, which was calculated according to standard curves created using standard samples provided by the kit manufacturer.

### RT-PCR

Total RNA was isolated from Jar cells and NK-92MI cells using TRIzol reagent following the manufacturer’s protocol (Invitrogen, USA). Using a reverse transcription kit (Takara, Japan), cDNA was synthesized from mRNA. GAPDH was used to normalize the mRNA expression levels. All primer sequences are listed in **Supplementary Table S1**.

### Western blotting

Proteins were extracted using RIPA buffer (Servicebio, China) containing protease inhibitors. Protein lysates (30 µg) were separated using 10% sodium dodecyl sulfate-polyacrylamide gels (SDS-PAGE) and subsequently transferred onto polyvinylidene fluoride membranes. The primary antibodies against HLA-F (Bioss #bs-17544R, 1:2000) and HLA-G (Thermo Fisher #PA5-98143, 1:2000) were used, along with biotinylated goat anti-rabbit IgG (H+L) secondary antibody (Abcam, UK). The antigen-antibody reaction was visualized by enhanced chemiluminescence assay (ECL; Thermo Fisher, USA).

### Statistical analysis

SPSS (v22, IBM, Armonk, NY) was used for statistical analysis, and P-values < 0.05 were considered statistically significant. Comparisons were performed using χ^2^ tests, paired t-tests, or two-sided Wilcoxon Rank Sum tests as appropriate.

## Results

### Single-cell RNA sequencing reveals cell heterogeneity in preeclamptic and control decidua

**Figure 1:**
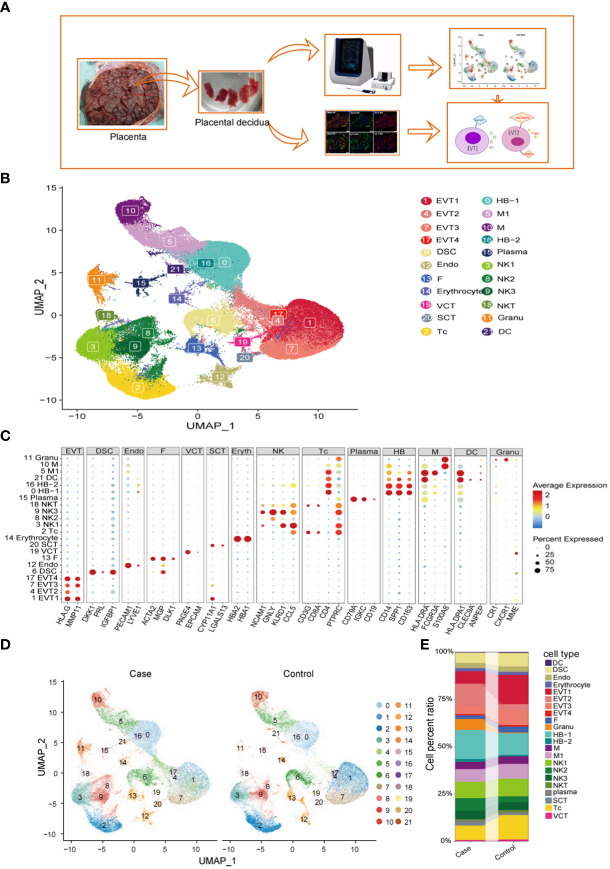
Identification of cell types at the maternal-fetal interface in preeclampsia and normal individuals. **(A)** Workflow depicting placental decidual sampling method and single-cell transcriptome profiling. **(B)** Placental decidual cell clusters from BD Rhapsody and Illumina HiSeq 4000 scRNA-seq analysis visualized by UMAP. **(C)** Dot plots showing markers of different cell clusters. **(D)** Cell distribution in Case (preeclampsia) and Normal control groups. **(E)** Sankey diagram showing cell percent ratio between groups.

Decidual tissues obtained from the placentas of two patients with PE (case group) and two women with normal pregnancies (control group) were lysed to prepare single-cell suspensions. Single cells were captured using nano-wells and the BD Rhapsody platform and used to generate single-cell transcriptome libraries, which were then sequenced on an Illumina HiSeq 4000 apparatus (**Figure 1A**). For details on the patients in the case group and the control group, see **Supplementary Table S2**. After preliminary quality control, a total of 101,250 cells were included in the analysis, including 54,776 from the case group and 46,474 from the control group. We then analyzed variations in gene expression across all cells and grouped them based on the gene expression profiles of known cell-type markers; in this way, we identified 22 cell clusters and 14 cell populations (**Figures 1B, C**, **Supplementary Figures S1A, B**). HBs, monocyte/macrophages, EVTs, and NK cells accounted for the majority of the cell population. Compared to control samples, preeclamptic decidua had higher proportions of HBs, neutrophils, EVT2, and NK2 cells, but lower proportions of EV1, EVT3, and cytotoxic T lymphocyte (Tc) cells (**Figures 1D, E**). For the complete list of DEGs between the case group and the control group, see **Supplementary Table S3**.

### Detailed classification and trajectories of myeloid cells at the MFI

**Figure 2:**
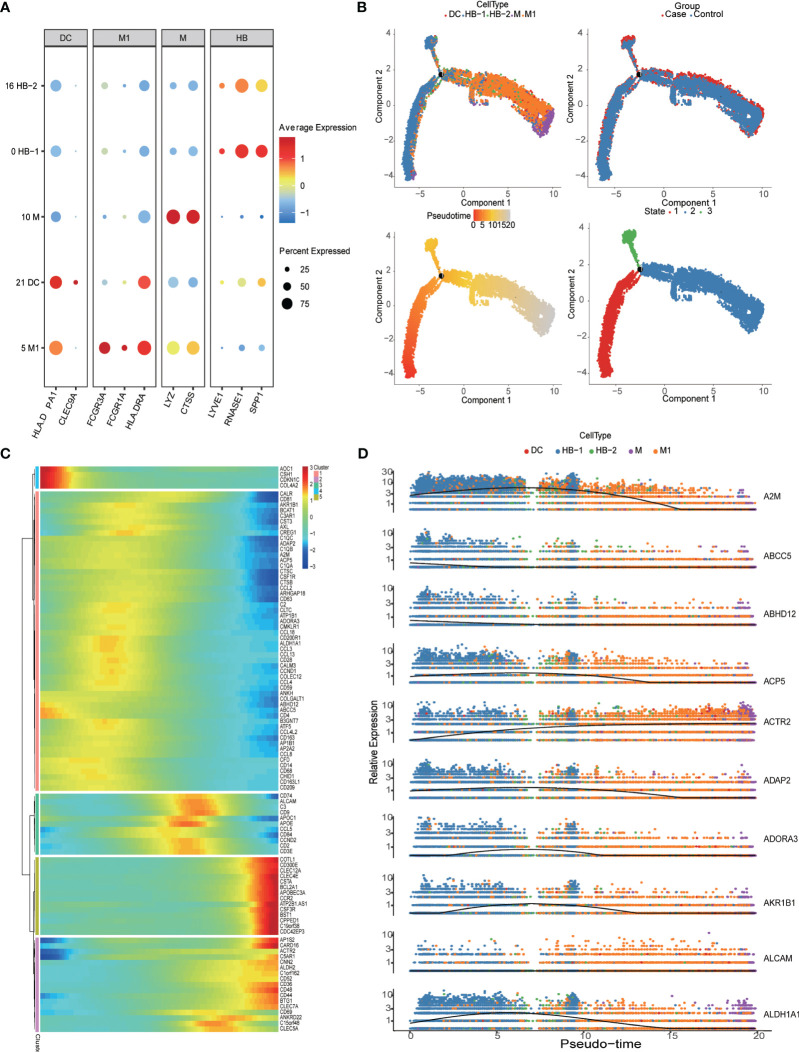
Detailed classification and trajectories of myeloid cells in the maternal-fetal interface. **(A)** Dot plots showing markers of different myeloid cell populations in the maternal-fetal interface. **(B)** Trajectory distribution of each myeloid cell population over time in different groups with the developmental starting point on the left and the developmental endpoint on the right. **(C)** Heatmap showing clustering genes by pseudo-temporal expression pattern. **(D)** The trajectory of some typical genes.

Monocytes/macrophages were the predominant population of immune cells at the MFI during the third trimester. The PE group had a total of 15,200 HLA-DRA^+^CD14^+^ myeloid cells, representing 43.65% of the total CD45+ immune cell population, while the control group contained a total of 11,378 myeloid cells, accounting for 43.3% of immune cells. Within this group, we analyzed the specific characteristics of different myeloid subtypes. Clusters 0 and 16 were LYVE1^+^SPP1^+^CD163^+^ M2-like fetal macrophages, i.e., HBs; Cluster 5 was LYVE1^-^CD16^+^ M1 macrophages (M1); Cluster 10 was HLA-DRA^+^CD14^+^CD86^+^S100A8^+^, suggested that it was derived from monocytes (M); and Cluster 21 was HLA-DPA1^+^CLEC9A^+^ dendritic cells (DCs) (5, 27) (**Figures 1C**, **2A**). A pseudo-time analysis of developmental trajectories in the myeloid cell population at the MFI showed that HB-1 cells (cluster 0) were in the early stage of myeloid differentiation, HB-2 cells (cluster 16) were in the middle stage, and M and M1 cells were in the late stages of myeloid cell differentiation (**Figure 2B**). We then selected some typical genes in the cell development trajectory for further analysis. Genes such as *A2M*, *ABCC5*, *ABHD12*, and *ACP5* were active in the early stage of myeloid differentiation, while *ACTR2* was expressed in the late stage of myeloid differentiation. Intermediate-stage marker genes such as *APOE*, *APOC1*, and *AKR1B1* may play a regulatory role in mononuclear macrophage differentiation (**Figures 2C, D**).

### Cell-to-cell communication at the maternal-fetal interface

**Figure 3:**
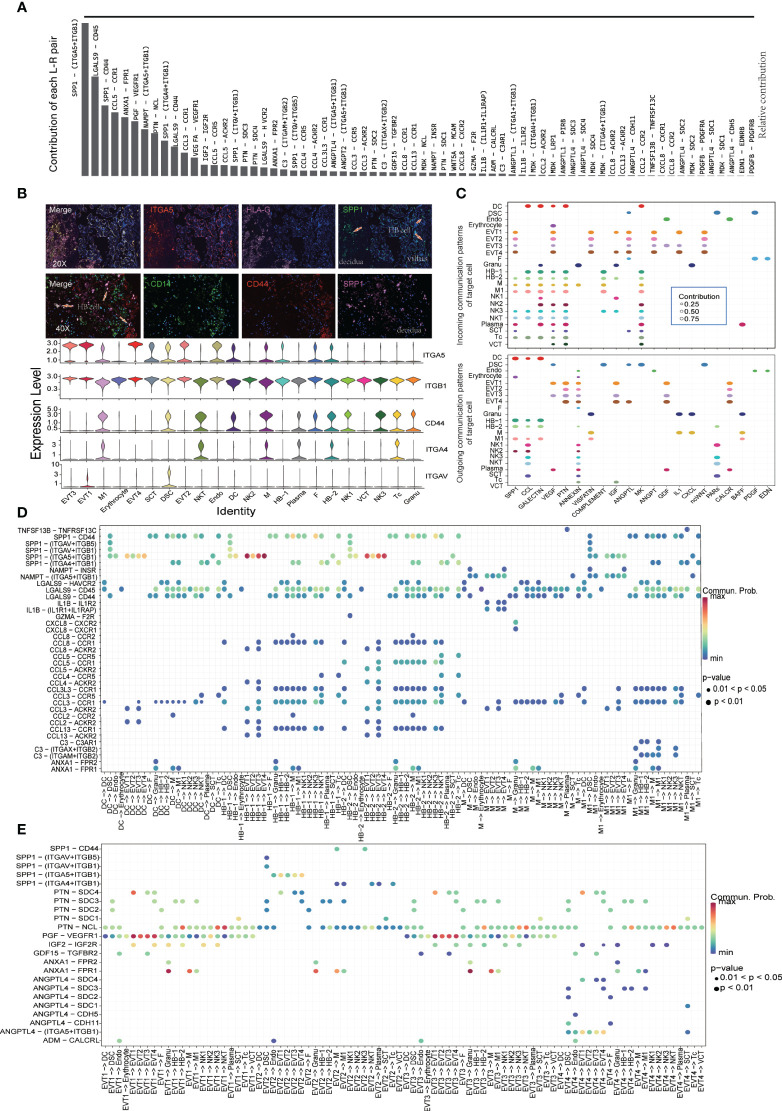
Cell-to-cell communication at the maternal-fetal interface shown by Cellchat. **(A)** Contribution of each L-R pairs at the maternal-fetal interface. **(B)** Immunofluorescence staining showing SPP1 and its receptor distribution. Arrows showing SPP1+ HB cell in villus and decidua. **(C)** Dot plot showing the incoming signaling patterns of targeting cells and outgoing signaling patterns of secreting cells. **(D)** Dot plot showing ligand-receptor interactions between myeloid cells and other cell populations. **(E)** Dot plot showing ligand-receptor interactions between EVT and other cell populations.

We used CellChat to construct a cell-cell communication network through an analysis of Ligand-Receptor (L-R) signaling exchanges at the MFI. **Figure 3A** shows the contribution of various L-R pairs, of which the top five were SPP1-(ITGA5+ITGB1), LGALS9-CD45, SPP1-CD44, CCL5-CCR1, and ANXA1-FPR1. Immunofluorescence staining revealed that intervillous and decidual CD14^+^SPP1^+^ HBs interact with ITGA5 on the surface of HLA-G^+^ EVTs and CD44 on the surface of other immune cells (**Figure 3B**). Chemokines such as CCL5 and CCL3, which are secreted by immune cell populations other than M, interact extensively with immune cells other than NK1, NK2, and plasma populations. Instead, the predominant cell clusters that secrete VEGF were DSC, EVT1, EVT3, and SCT. Its ligand FLT1 was widely expressed on trophoblasts, leukocytes, and lymphocytes, with the exception of DSC, endothelial cells (Endo), fibrocyte (F), and granulocyte (Granu) (**Figure 3C** and **Supplementary Figures S2A, B**).

Our analysis of L-R signal exchanges between subtypes of monocyte/macrophages and other cell populations found the strongest cross-talk between SPP1^+^ HBs and ITGA5^+^ EVT1/4 (**Figures 3B, D**). Unlike HB-1, HB-2 can secrete CCL5, which communicates with ACKR2^+^ EVT1/3 cells and interacts with CCR1^+^ or CCR5^+^ monocytes, macrophages, and NK cells (**Figure 3D**). EVT1 and EVT3 cells had strong secretion of PGF, IGF2, GDF15, and other cytokines that promote placental growth and development. IGFBP1^+^SPP1^+^ EVT2 cells, like monocyte/macrophages, demonstrated extensive cross-talk with other ITGA5^+^ EVTs, ITGAV+ DSC, and ITGA4^+^ or CD44^+^ immune cells, while their secretion of PTN and other growth factors was relatively weak (**Figure 3E**). In PE samples, upregulated expression of SPP1 at the MFI mainly originated from the M/M1 and EVT cell populations (**Supplementary Table S3**); there was thus enhanced cell-crosstalk between IGFBP1^+^SPP1^+^ EVT2, SPP1^+^ M1, and their receptor cell populations in these tissues.

### The MFI in PE patients contains an overabundance of EVT2 cells characterized by features of inflammatory fibrosis

**Figure 4:**
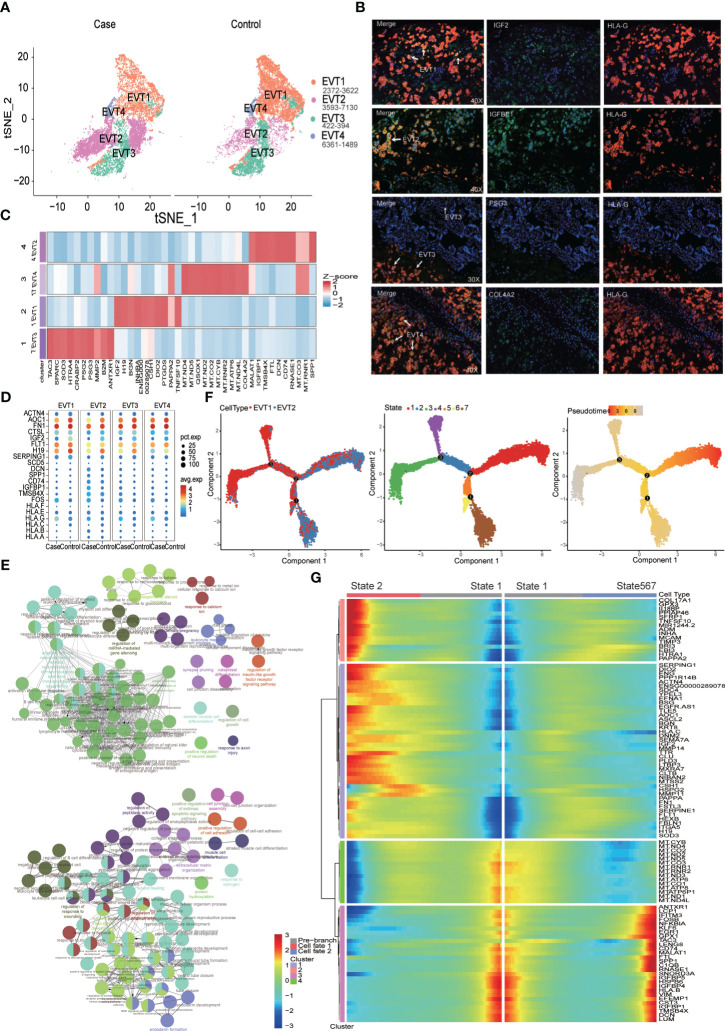
Increased proportion of CD74+IGFBP1+EVT2 cells at the maternal-fetal interface in preeclampsia individuals. **(A)** EVT Cell distribution in Case (preeclampsia) and Normal control groups. **(B)** Immunofluorescence staining showing IGF2+EV1, IGFBP1+EVT2, PSG3+EVT3 and COL4A2+EVT4. **(C)** Heatmap showing top 10 maker genes of different EVTs subtypes. **(D)** Dot plot showing some DEGs distribution in Case and Control groups. **(E)** GO analysis of the top 100 up- (up) and down- (down)-regulated DEGs of the EVT2 subtype. Nodes represent terms and edges represent connections with the color and size reflecting the enriched classification and statistical significance, respectively. Only terms with P less than 0.05 are shown. **(F)** Trajectory distribution of EVT1 and EVT2 cell population over time with the developmental starting point on the right and the developmental endpoint on the left. **(G)** Differentially expressed genes before and after branching point 2.

The MFI contains a large number of EVTs; in this study, a total of 19,389 EVT cells were captured for analysis. In all case and control samples, EVT cells could be divided into four subtypes (**Figures 4A-C**). Of these, EVT2 cells were overrepresented in the MFI of PE patients compared to the control samples (**Figure 4A**). When we examined DEGs in EVTs between the case and control group (**Supplementary Table S3**) and subjected the resulting DEGs to KEGG pathway analysis (Kyoto Encyclopedia of Genes and Genomes; **Supplementary Table S4**), we found that EVT cells in PE patients demonstrated abnormalities in signaling pathways such as antigen processing and presentation, fluid shear stress and atherosclerosis, complement and coagulation cascades, apoptosis, focal adhesion, and Rap1 signaling. Gene expression levels in related signaling pathways were also unevenly distributed among the four EVT subtypes (**Figure 4D**).

The proportion of EVT2 subtypes with elevated expression of the *MALAT1*, *IGFBP1*, *CD74*, *RNASE1*, *HLA-B*, and *SPP1* genes was significantly higher in the MFI of PE patients than in controls (**Figures 4A-D**). *CD74*, *RNASE1*, and *SPP1* are macrophage marker genes, while *IGFBP1* is a fibroblast marker gene (**Supplementary Figures S1A, B**); this pattern suggests that there were inflammatory fibrotic changes in EVT2 cells. A Gene Ontology (GO) enrichment analysis of the top 100 DEGs between EVT2 and other EVT subtypes revealed that EVT2 cells demonstrated significant downregulation in functions related to angiogenesis, wound healing, cell adhesion, and extracellular matrix formation, and upregulation related to antigen presentation, adaptive immunity, and response to steroid hormones (**Figure 4E** and **Supplementary Tables S5**, **S6**). Instead, EV1 cells were mainly associated with negative regulation of leukocyte differentiation and the promotion of placenta formation (**Supplementary Figures S3A-F**). Further pseudo-time analysis was performed on the overall EVT1 and EVT2 subtypes to identify the key genes in the conversion of EVT2 to EVT1. This indicated that there were three branch points of cell development between EVT1 and EVT2, this process involved a total of seven cell states, and EVT2 was closer to the developmental starting point (**Figure 4F**). An analysis of differentially expressed genes before and after developmental branch point 2 showed that the transition from STATE1 to STATE2 was closer to the developmental shift from EVT2 to EVT1 (**Figures 4F, G**). Markers of the intermediate stage included the genes *CSH1*, *HSPG2*, and *MMP11*, which may play an important role in regulating EVT2 differentiation.

### Ribosomal functions of NK cells are significantly reduced in patients with PE

**Figure 5:**
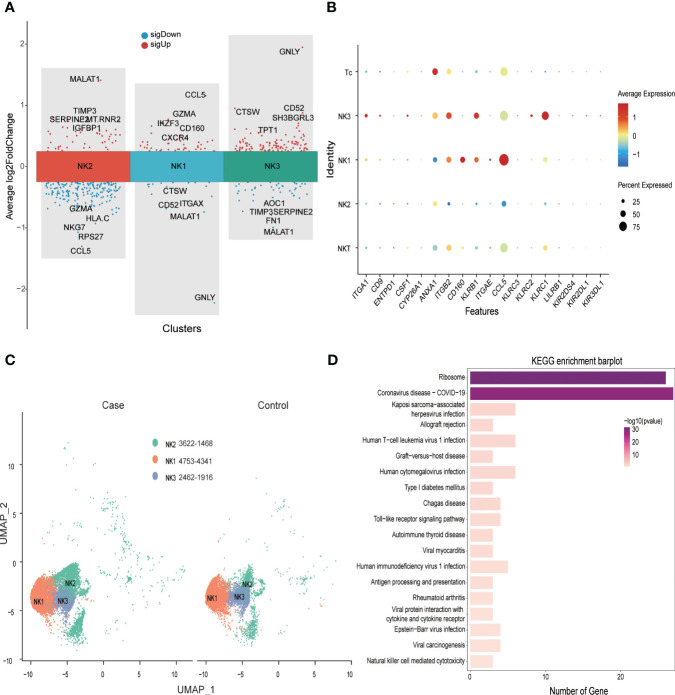
Significantly down-regulated NK cell ribosome pathway at the maternal-fetal interface of preeclampsia. **(A)** Volcano plot showing top 5 sigUp and sigDown genes of different NK subtypes. **(B)** Dot plot showing some makers’ distribution of NK and T populations. **(C)** NK cell distribution in Case (preeclampsia) and Normal control groups. **(D)** Bar plot showing KEGG enrichment analysis of DEGs of NK cell in Case and Control groups.

In the first trimester, decidual NK cells account for 70% of immune cells, but at the MFI in the third trimester, NK cells represented 32% of CD45+ immune cells in the PE group and 31% in the control group. We identified three decidua NK subtypes (NK1, NK2, and NK3) at MFI in the third trimester. Unlike predominant NK cells in the first trimester, which secrete CSF1 (5), NK1 cells in the third trimester mainly secreted chemokines such as CCL5, CCL4, and CCL3, and expressed IKZF3, GZMA, and CD160. The NK2 subtype was characterized by the expression of SNORD3A, MALAT1, TIMP3, and SERPINE2, and NK3 was marked by GNLY, CD52, and CTSW (**Figure 5A**). In terms of receptor expression, the NK3 subtype had the highest levels of NKG2A and NKG2C and slightly higher levels of KIR receptors and CSF1 than the other NK cell subtypes (**Figure 5B**).

Compared with control samples, patients with PE exhibited a disproportionately high number of NK2 subtypes at the MFI (**Figure 5C**). Analysis of differential gene expression in NK cells (**Supplementary Table S3**) revealed that a variety of MHC-I molecules were downregulated in PE patients compared to controls, with HLA-C being the most significant. These patients also evidenced decreased expression of CCL5 compared to controls, but slightly higher expression of CCL4 and CCL3. In addition, the expression of ribosome-related genes such as *RPS26*, *RPS27*, *RPL34*, and *RPS14* was significantly downregulated in PE patients. Analysis of KEGG signaling enrichment also revealed significant alterations in signaling pathways related to NK cell ribosomal functions, allograft rejection, and Toll-like receptors (**Figure 5D** and **Supplementary Table S7**).

### Pervasive inflammatory activation of monocyte/macrophages at the MFI in PE

**Figure 6:**
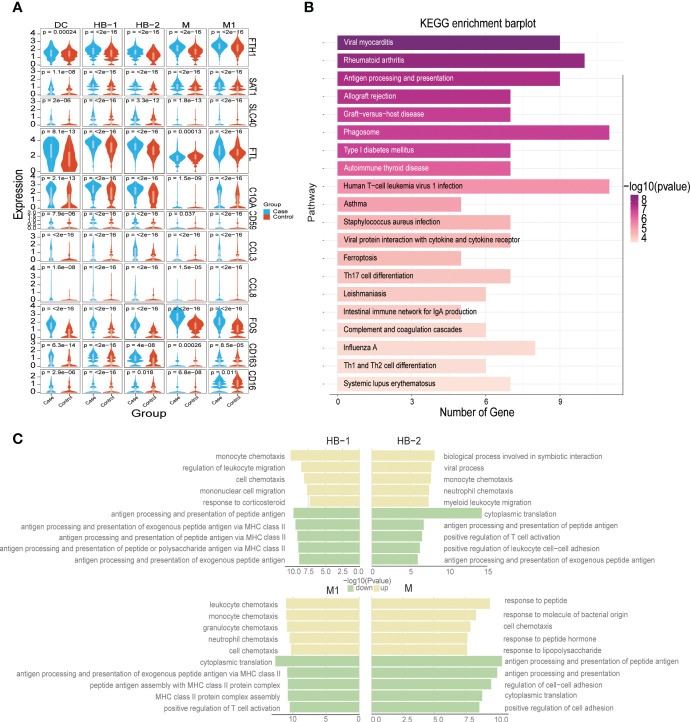
Dysfunction of myeloid populations at the maternal-fetal interface in preeclampsia. **(A)** Violin plot showing some DEGs distribution in Case and Control groups. **(B)** Bar plot showing KEGG enrichment analysis of DEGs of Hofbauer cell in Case and Control groups. **(C)** Five representative GO terms including increased and decreased enrichment listed based on NES (normalized enrichment score) and FDR (false discovery rate) between the PE and control groups in each myeloid cell subtype.

Hofbauer cells exhibited high levels of RNASE1, SPP1, CD14, and CD163 expression, and partial expression of LYVE 1 (**Figures 1C**, **2A**). We identified two subtypes of HB cells (HB-1 and HB-2) at the MFI in the third trimester, of which both were more abundant in samples from PE patients compared to controls (24.39% vs. 20.63% for HB-1 and 2.5% vs. 1.3% for HB-2, respectively) (**Figure 1E**). When we analyzed patterns of DEGs in HBs between patients with PE and healthy pregnant women (**Supplementary Table S3**), we found that PE samples exhibited upregulation of FTH1/SAT1/SLC40A1/FTL/HMOX1 and other members of the ferroptosis signaling pathway; FOS/FOSB, CCL3, CCL4, and other components of the TLR signaling pathway; and C1QA/C5AR1/CD59/A2M/SERPINE1/SERPINE2 and other genes related to complement and coagulation cascade signaling pathways. Instead, we observed significant downregulation of AOC1, H19, FN1, HLA-DQA1, MT, RNR1, and CD74 (**Figure 6A**). KEGG pathway enrichment analysis revealed that HBs in the PE group demonstrated abnormalities in signaling pathways associated with viral myocarditis, rheumatoid arthritis, antigen processing and presentation, allograft rejection, graft-versus-host disease, and ferroptosis (**Figure 6B**, **Supplementary Table S8**). Furthermore, between the control group and the PE group, analysis of GO enrichment of DEGs highlighted that, in the PE samples, HB-1 cells demonstrated enrichment in functions related to monocyte chemotaxis, but downregulation in those associated with antigen presentation; HB-2 cells exhibited upregulation in biological processes involved in symbiotic interactions and downregulation in cytoplasmic translation; monocytes had an upregulated response to peptides but downregulated antigen processing and presentation of peptide antigens; and, finally, M1 macrophages evidenced upregulation in leukocyte chemotaxis, monocyte chemotaxis, and other similar processes, and downregulation in cytoplasmic translation (**Figure 6C**).

### The synergistic effect of HLA-F and HLA-G in maternal-fetal immune tolerance

**Figure 7:**
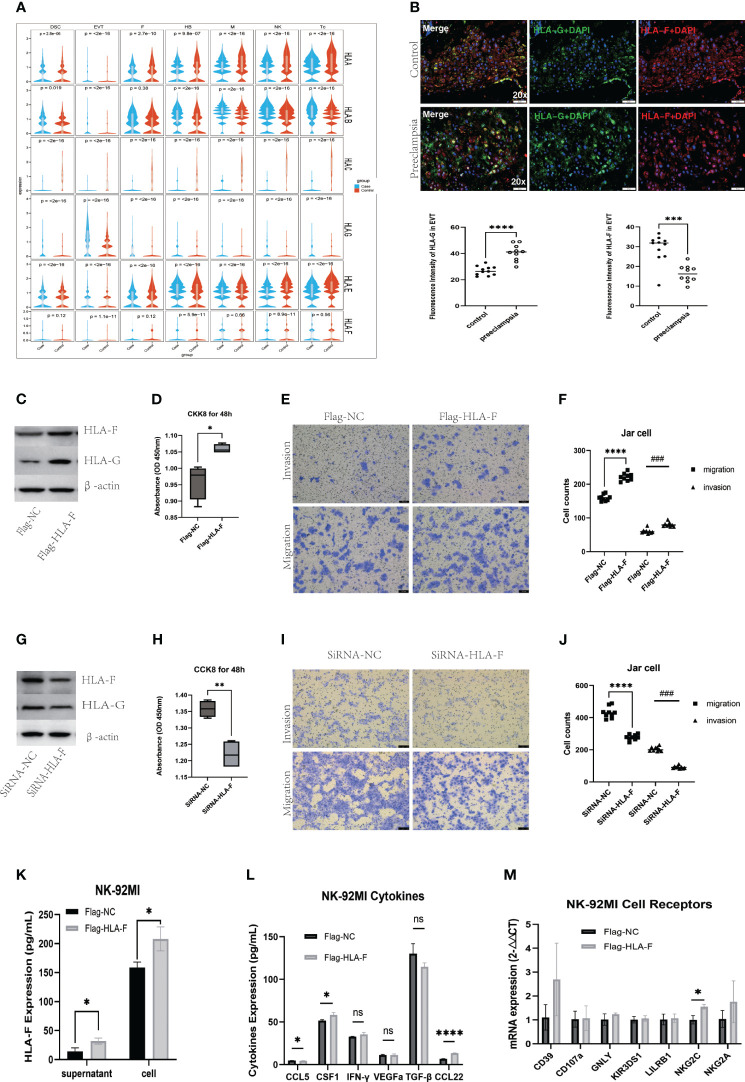
Augmented immune tolerance at the maternal-fetal interface under collaboration of HLA-F and HLA-G. **(A)**Violin plots showing abnormal expression of multiple HLA genes in several kinds of cells. **(B)** Immunofluorescence staining analysis showing increased HLA-G and decreased HLA-F expression in EVT cells of preeclampsia. **(C)** Western blot verifying the overexpression of HLA-G in Jar cells induced by the overexpression of HLA-F via plasmid transfection. **(D)** CCK8 assay showing cell viability of Jar cells with overexpressed HLA-F. **(E-F)** Transwell invasive and migration assay of Jar cells with overexpressed HLA-F. **(G)** Western blot demonstrating reduced expression of HLA-G in Jar cells with inhibited expression of HLA-F via siRNA transfection. **(H)** CCK8 assay showing cell viability of Jar cells with the knockdown of HLA-F. **(I-J)** Transwell invasive and migration assay of Jar cells with knockdown HLA-F. Bar plots presenting overexpressed HLA-F in NK-92MI cell supernatant and cells **(K)**, increased CSF-1 and CCL22 secretion in NK-92MI cells under the overexpressed HLA-F via ELISA assay **(L)** and increased NKC2C mRNA expression in NK-92MI cells with overexpressed HLA-F via RT-PCR **(M)**. Data are presented as the mean ± SD. All the cell function experiments, ELISA assay and RT-PCR were repeated three times. Statistical significance was assessed by unpaired Student’s t-test; ns, no statistical significance; (*P < 0.05, **P<0.01, ***P<0.001, ****<0.0001, ^###^P<0.001).

Single-cell RNA sequencing revealed alterations in patterns of HLA expression in various cell subsets at the MFI in patients with PE (**Figure 7A**). An analysis of specific cell subtypes indicated that HLA-C was significantly downregulated in Tc, monocyte/macrophages, and NK cell subtypes; HLA-G was significantly upregulated in EVT subtypes; and HLA-B expression was upregulated in lymphocyte subtypes and EVT2 subtypes (**Figure 4C**). HLA-F was mainly expressed in immune cell populations (**Supplementary Table S9**, **Supplementary Figure S4**), and of little difference in mRNA expression between case and control groups (**Figure 7A**, **Supplementary Table S3**).

We used dIF to verify the expression of HLA-G and HLA-F in placental decidua obtained from 10 PE patients and 11 healthy controls (**Supplementary Table S10**). At the MFI of PE patients, the expression of HLA-G proteins in EVTs was significantly upregulated compared to controls, while HLA-F in HLA-G+EVTs was significantly downregulated (**Figure 7B**). The function of HLA-G in trophoblast cells has been studied extensively, but relatively little is known about HLA-F. To investigate this further, we used human placental choriocarcinoma Jar cells and NK-92MI cell lines to study the function of HLA-F in trophoblasts and lymphocytes, respectively. After transfecting Jar cells with an HLA-F overexpression plasmid, levels of HLA-F protein and HLA-G protein were both increased significantly when compared to Jar cells transfected with the control plasmid (**Figure 7C**), along with the proliferation, invasion, and migration of trophoblast cells (**Figures 7D-F**). The transfection of Jar cells with HLA-F siRNA led to a significant decrease in the expression of both HLA-F and HLA-G (**Figure 7G**), and a significant decline in the proliferation, migration and invasion ability of trophoblast cells (**Figures 7H-J**). When NK-92MI cells were transfected with the HLA-F overexpression plasmid, the expression of HLA-F proteins in both the cell supernatant and cell suspension increased, along with the expression of the CSF1 and CCL22 cytokines and the NKG2C receptor in NK cells, while the secretion of CCL5 decreased (**Figures 7K, L, M**).

## Discussion

Preeclampsia is a complex multisystem disease, diagnosed by sudden-onset hypertension, proteinuria, and other signs of maternal and fetal damage manifested after 20 weeks of gestation (28). Severe preeclampsia is diagnosed based on further elevation of blood pressure (systolic ≥160 mm Hg or diastolic of ≥100 mm Hg) or any of the following: thrombocytopenia, impaired liver function, progressive renal insufficiency, pulmonary edema, and the onset of cerebral or visual disturbances that may end in HELLP (hemolysis, elevated liver enzymes, and low platelet count) syndrome and/or eclampsia (29). Early-onset preeclampsia is a clinical symptom manifested before 34 week (1), which generally have more severe clinical presentation in the mother and is often associated with EVT maldevelopment in the immune aspect (30). The immunohistochemical study found more CD68+ monocytes/macrophages and myeloperoxidase (MPO)+ Granu in the placenta of the “immunological” preeclampsia subtype (31). Otherwise, inadequate extravillous trophoblast invasion and spiral artery remodeling were more often observed in the preterm preeclampsia subtype (28). On the other hand, gene modules contributing to maternal or fetal diseases were also revealed under placenta transcriptomics of women with early-onset preeclampsia, such as *LEP*, *PAPPA2*, *FLT1* to maternal blood pressure, and *KIT*, *FBLN1*, *HSD17B1, CSH1* to fetal birth weight, and with late-onset preeclampsia, such as *PAPPA*, *ADAM12*, *CYP19A1*,*CSH1*, *PSG3* to placental abnormal function (30, 32), the differences of which under different subtypes of preeclampsia reflected the heterogeneous etiology of this disease (28, 32).

In this study, we analyzed samples of placental decidua from women with healthy pregnancies and those diagnosed with PE before 37weeks. Single-cell sequencing analysis revealed that these tissues, taken from the MFI, contained numerous cells of fetal origin, such as HB cells and EVTs, along with cells of maternal origin such as DSC s, NK cells, and monocyte/macrophages, among others. We examined these cell populations with the goal of exploring the mechanisms of immune disturbances at the MFI in PE; specifically, we assessed abnormal proportions of cell subtypes, functional irregularities, and alterations in cell communication and differentiation at the MFI compared to healthy samples. These data were used to construct a panoramogram of immune disturbances at the MFI in PE patients.

In the third trimester of pregnancy, monocytes/macrophages are the most common immune cells at the MFI. In PE patients, the main changes we observed were related to M1 polarization due to increased ferroptosis, the release of inflammatory factors resulting from activation of the TLR pathway, the activation of cytotoxic T/NK cells due to abnormal antigen presentation, *in situ* damage at the MFI, and damage to maternal systems caused by increased complement release. Our pseudo-time analysis revealed that *AKR1B1*, *APOC1*, *APOE*, and other genes characteristic of intermediate developmental stages may have important regulatory roles in monocyte/macrophage differentiation. Previous studies have shown that inhibition of *APOC1* transcription can promote the transformation of M2 macrophages to the M1 type via the tumor-suppressing activity of the ferroptosis pathway (33). It may be interesting to investigate if regulating *APCO1* and other genes to reverse M1 monocytes/macrophages could help in the treatment of PE. In patients with PE, the most downregulated gene in HB cells was *AOC1*, but little is known about the role of this gene in macrophage differentiation and polarization and in the pathogenesis of PE. To date, *AOC1* has been reported to promote the epithelial-mesenchymal transition (EMT) in gastric glandular cells and gastric carcinogenesis (34), and to inhibit cancer cell proliferation in prostate cancer (35). The expression changes in *AOC1* in HBs of patients with PE merit further study.

During the first trimester, NK cells at the MFI are characterized by the secretion of CSF1 and the expression of KIR and LILRB1 receptors, which play an important role in promoting embryonic growth and facilitating maternal immune tolerance (5). However, the current study found that, in the third trimester, NK cells at the MFI mainly secreted CCL5, which interacted with CCR1^+^ NK cells and monocyte/macrophages, and CCR5^+^ Tc and NKT cells. Previous studies have shown that increasing expression levels of CCL5 in HTR-8/SVneo cells (via transfection with lncRNA uc003fir) can enhance trophoblast invasion and migration (36). However, other studies have reported that, by upregulating CCL5 expression, oncolytic viruses can recruit NK cells to induce tumor regression (37). High levels of CCL5-CCR5 are observed in the malignant tumor microenvironment (38), while sharing certain similarities in growth and behavior with trophoblasts and, indeed, the MFI. It is therefore likely that CCL5 plays a role in promoting immune tolerance at the MFI, and that a reduced proportion of CCL5-secreting NK1 cells in PE contributes to immune dysregulation. However, the most notable signaling pathway change in NK cells in patients with PE was the downregulation of the ribosomal pathway, which is known to be involved in juvenile dermatomyositis and is associated with autoimmune diseases (39). The significance of this change in the context of PE requires further study.

Our current study captured 19,389 EVT cells at the MFI, which is the largest number of these cells to be analyzed in a single-cell transcriptome sequencing study of PE thus far. Among these, there was a significant overrepresentation of the EVT2 subtype in PE samples compared to controls, and these cells exhibited relatively high expression of genes associated with inflammatory fibrosis, such as *MALAT1*, *IGFBP1*, *CD74*, *RNASE1*, and *SPP1*. These findings were partly supported by a single cell-seq of decidual tissue of the placental bed that, SPP1 was found up-regulated in EVTs of late-onset preeclampsia (40). Likewise, the level of IGFBP1, an important marker of decidualization (41), in serum of preeclampsia patients was significantly lower than that of normal pregnant women before the onset of clinical symptoms but was reversed in late pregnancy (42). On the other hand, IGFBP1overexpression inhibited the proliferation, invasion, migration, and apoptosis of HTR-8/SVneo trophoblast cell line (43). Though elevated decidual and plasm IGFBP1seems to be a protected maker in the early gestation on the perspective of decidualization (44), at the third trimester, over-expression of IGFBP1 might be a symptom of dysfunctional trophoblast. Thus, the SPP1+IGFBP1+ EVT2 cells might contribute to inflammatory fibrosis in the PE placenta and may play an important role in the pathogenesis of PE.

In contrast, the EVT1 subtype, characterized by the expression of *IGF2*, *H19*, *FTL1*, and other genes that promote placental formation, was predominant in normal pregnant women. Pseudo-time analysis of the EVT1 and EVT2 subtypes suggested that *CSH1* and *HSPG2* may regulate EVT differentiation, and both are downregulated in preeclamptic EVT, in line with previous placenta transcriptomics of women with both early-onset and late-onset preeclampsia (30, 32). *CSH1* is located in a growth hormone locus on chromosome 17; mutation or deletion of this gene is associated with placental prolactin deficiency and the pathogenesis of Silver-Russell syndrome (45), but its role in regulating EVT differentiation and the pathogenesis of PE is unclear. Instead, *HSPG2* is associated with tumor progression and poor cancer prognosis (46). Mutations in this gene can increase a tumor’s responsiveness to immune checkpoint inhibitors and improve the efficacy of targeted drug therapy (47), but again, there is a lack of basic research on the relationship between this gene and the pathogenesis of PE. Another obvious change in EVTs from the PE group compared to control cells was the downregulation of *IGF2* (41–44). *IGF2* and its lncRNA *IGF2-AS*-encoded peptide are important for trophoblast proliferation (48, 49). However, further study is needed to decipher the mechanisms of these changes in EVTs in patients with PE.

Previous studies on the relationship between HLA and PE have mostly focused on HLA-G, although recent work reported that HLA-C is downregulated in trophoblast cells of PE patients (50), and that the combination of maternal KIR AA genotype and fetal HLA-C2 genotype increases the risk of PE (51–53). In general, though, the specific role of HLA-C in EVT differentiation and immune regulation at the MFI is still unclear. HLA-G has been reported to play an important role in regulating maternal-fetal immune tolerance (54–56), but the described levels of placental HLA-G expression in PE patients have been inconsistent (50, 56, 57). Here, we found significantly higher levels of HLA-G protein in patients with PE, which may be related to the earlier gestational age at which pregnancy ended (and thus when the samples were taken) for the PE patients. Another explanation could be that the increased expression of HLA-G acts as a compensation mechanism for the inflammatory activity present at the MFI in patients with PE.

Recently, an increasing number of studies have explored the role of HLA-F in immune tolerance at the MFI. Several have found that polymorphism in the *HLA-F* gene may affect the expression of HLA-F at the MFI, with repercussions for the implantation success rate in ART treatment (24, 58). The present study supports a previous finding of reduced HLA-F expression in EVTs of PE patients (50). It is believed that, since *HLA-F* and *HLA-G* share certain eQTLs in common, these two genes may act in concert. Here, we observed clear regulation of HLA-G by HLA-F. The most important transcription factors of *HLA-F* are NF-κB and IFN-γ (59). Thus, increased levels of NF-κB and IFN-γ lead to increased HLA-F expression in EVTs and, consequently, increased downstream HLA-G expression during the inflammatory activation at the MFI. Both HLA-F and HLA-G work together to maintain immune tolerance and regain a stable state by binding to inhibitory receptors of NK cells or T cells. Defects in this compensation mechanism, and the associated reduction in HLA-F expression, may contribute to the uncontrolled inflammatory response associated with the onset of PE.

This study also found that HLA-F can promote the invasion, migration, and proliferation of trophoblast cells. In addition, when HLA-F was overexpressed in NK cells, we observed increased expression of the activating receptor NKG2C and the cytokines CSF1 and CCL22. CSF1 can recruit and transform macrophages to the M2 phenotype to promote immune escape in a tumor microenvironment (60), and can increase invasion of EVT (61), suggesting that it may possibly participate in the establishment of maternal-fetal immune tolerance in early pregnancy. Indeed, decreased placental expression of CSF1 has previously been observed in PE patients and might be associated with PE pathogenesis (62). CCL22 is known to recruit Treg cells and promote immunosuppression in tumor immune microenvironment (63–65), which might have important implications for maternal-fetal immune tolerance. Pregnancy-trained decidual memory NKG2C^+^ NK cells have increased production of CSF1 and VEGFa and might be involved in proper placentation (66). In sum, our results suggest that HLA-F and HLA-G may promote immune homeostasis at the MFI by promoting EVT proliferation and migration and regulating the immune function of NK cells.

From the perspective of decidualization, glycolytic activity in DSC was reported to indicate the degree of decidualization (67), glycolysis-related DEGs examined by single cell-seq in decidual core cells from late-onset PE patients were uncovered (40), as well as reduced expression of hexokinase 2 that suppresses glycolysis impaired the decidualization (68). Since it has been reported that the down-regulation of *HLA-F* expression can reduce the hexokinase 2-dependent glycolysis (69), we speculate that the reduced HLA-F expression may contribute to the abnormal decidualization via the glycol-metabolic pathway, even though the effect of HLA-F on glycolytic activity still needs further examination in DSCs and other cells at MFI.

Previous single-cell transcriptome sequencing studies in patients with PE have mostly focused on placental tissue (70, 71). Zhou et al. found that EVT in patients with PE can be divided into four subtypes, but the number of EVT cells in that study was low (25). Here, we wanted to focus on the MFI and thus collected decidual tissue from the placental maternal surface, and the number of EVT cells captured was large. Despite this, there are still certain limitations of this work. First, the overall number of cases was small, and there may have been differences in sampling and gestational age between samples. Second, because of the high cell activity requirements for single-cell sequencing, differences between batches and in sequencing depth in the preparation of the single-cell suspensions could also represent potential sources of statistical error.

In conclusion, by analyzing scRNA-seq results obtained from the MFI of patients with PE and those with healthy pregnancies, this study revealed abnormalities in cellular functions, communication, and differentiation in PE. In particular, we noted alterations in maternal-fetal immune cells such as HB cells, NK cells, monocyte/macrophages, and fetal EVT cells. We detected disruptions in EVT–immune cell cross-talk caused by widespread abnormal expression of HLA molecules at the MFI, which may play a fundamental role in the pathogenesis of PE. In addition, this study suggests that HLA-F works together with HLA-G as an important promoter of maternal-fetal immune tolerance, and the abnormal expression of these two genes may affect the establishment of this tolerance and contribute to pregnancy pathologies such as PE.

## Data availability statement

The datasets presented in this study can be found in online repositories. The names of the repository/repositories and accession number(s) can be found below: HRA004699 (GSA).

## Ethics statement

The studies involving humans were approved by Ethics Committee of Sichuan Provincial People’s Hospital. The studies were conducted in accordance with the local legislation and institutional requirements. The participants provided their written informed consent to participate in this study.

## Author contributions

FLu: designed the study, performed the experiments, performed the data analysis and statistical analysis and wrote the first draft of the manuscript. FLi: performed the experiments, performed the data analysis and statistical analysis and participated in the manuscript editing. YG: performed the experiments, performed the data analysis and statistical analysis and wrote the first draft of the manuscript. WX: edited the final version of the manuscript. YL: performed the clinical sample and data collection and patient recruitment. JYi: performed the clinical sample and data collection and patient recruitment. TF, SD, and HZ: provided technical consultation and participated in editing the final version of the manuscript. IH: revised earlier version of the manuscript. XL: conceived the original idea and editor earlier version of the manuscript. YH: designed and supervised the study and edited the first draft of the manuscript. JYu: conceived the original idea, designed the study and edited the final version of the manuscript. All authors contributed to the article and approved the submitted version.

## Supplementary material

Supplementary Figure 1 Heatmap **(A)** and Volcano plot **(B)** show makers of each cell cluster.

Supplementary Figure 2
**(A)** Dot plot showing ligand-receptor interactions between NK cells and other cell population. **(B)** Dot plot showing ligand-receptor interactions between decidua stromal cells, endothelial cells and other cell population.

Supplementary Figure 3
**(A)** GO analysis of the top 100 up-regulated DEGs of the EVT1 subtype. **(B)** GO analysis of the top 100 down-regulated DEGs of the EVT1 subtype. **(C)** GO analysis of the top 100 up-regulated DEGs of the EVT3 subtype. **(D)** GO analysis of the top 100 down-regulated DEGs of the EVT3 subtype. **(E)** GO analysis of the top 100 up-regulated DEGs of the EVT4 subtype. **(F)** GO analysis of the top 100 down-regulated DEGs of the EVT4 subtype. Nodes represent terms and edges represent connections with the color and size reflecting the enriched classification and statistical significance, respectively. Only terms with P less than 0.05 are shown.

Supplementary Figure 4 HLA-F and some immune cell activating maker distribution in cell clusters.

Supplementary Table 1 Primer list for RT-PCR.

Supplementary Table 2 Patient information for single-cell RNA seq.

Supplementary Table 3 Overall DEGs between groups and DEGs for specific cell population between groups.

Supplementary Table 4 KEGG enrichment analysis of DEGs in EVT cells.

Supplementary Table 5 GO analysis of the top 100 up-regulated DEGs of the EVT2 subtype.

Supplementary Table 6 GO analysis of the top 100 down-regulated DEGs of the EVT2 subtype.

Supplementary Table 7 KEGG enrichment analysis of DEGs in NK cells.

Supplementary Table 8 KEGG enrichment analysis of DEGs in HB cells.

Supplementary Table 9 HLA-F expression in cell clusters.

Supplementary Table 10 Patient information for dIF.
