# Supplementary figures and images for: Single-cell profiling reveals immune disturbances landscape and HLA-F-mediated immune tolerance at the maternal-fetal interface in preeclampsia
Source: Front Immunol. 2023 Oct 3;14:1234577. doi: 10.3389/fimmu.2023.1234577 (PMC10579943; doi:10.3389/fimmu.2023.1234577)

0 1 2 3 4 5 6 7 8 9 10 11 12 13 14 15 16 17 18 19

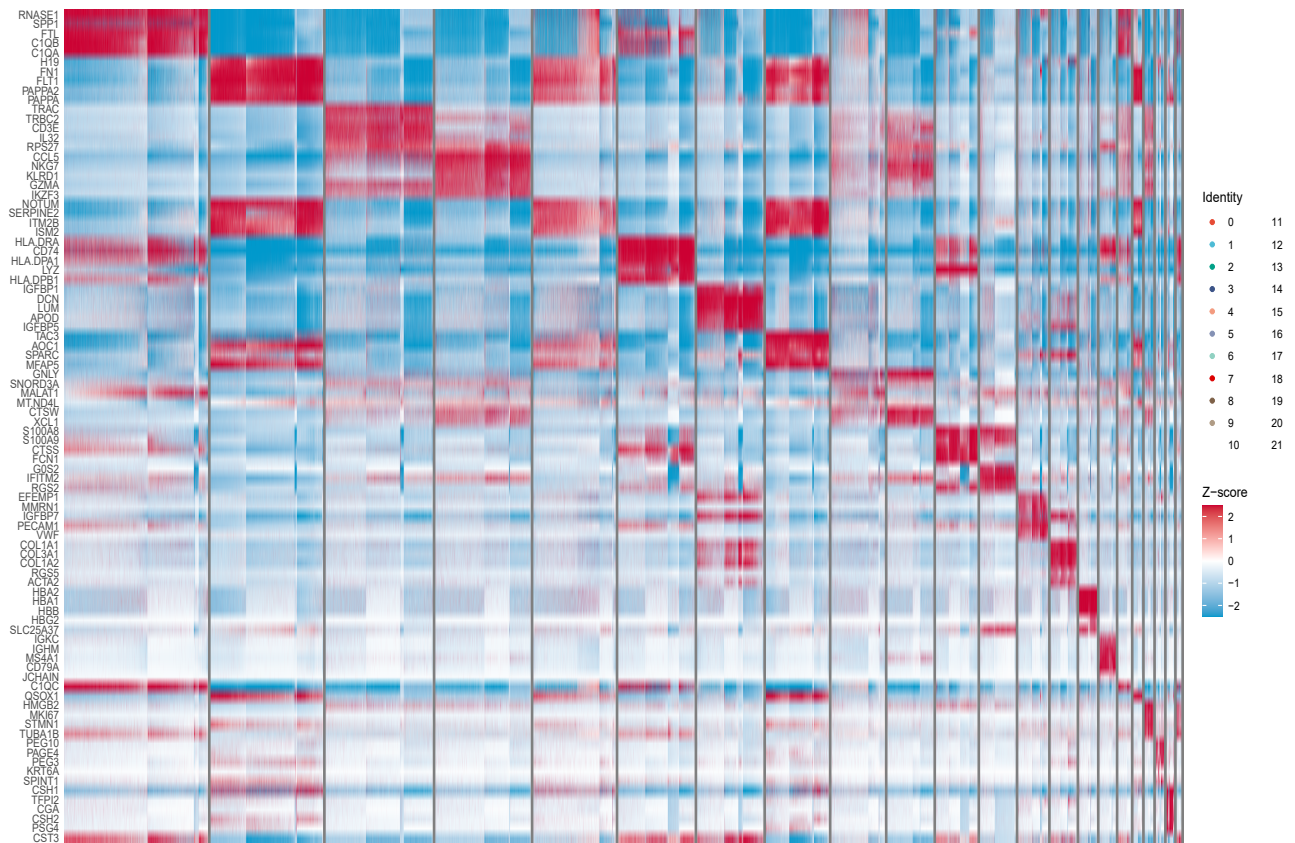

0 1 2 3 4 5 6 7 8 9 10 11 12 13 14 15 16 17 18 19 20 21

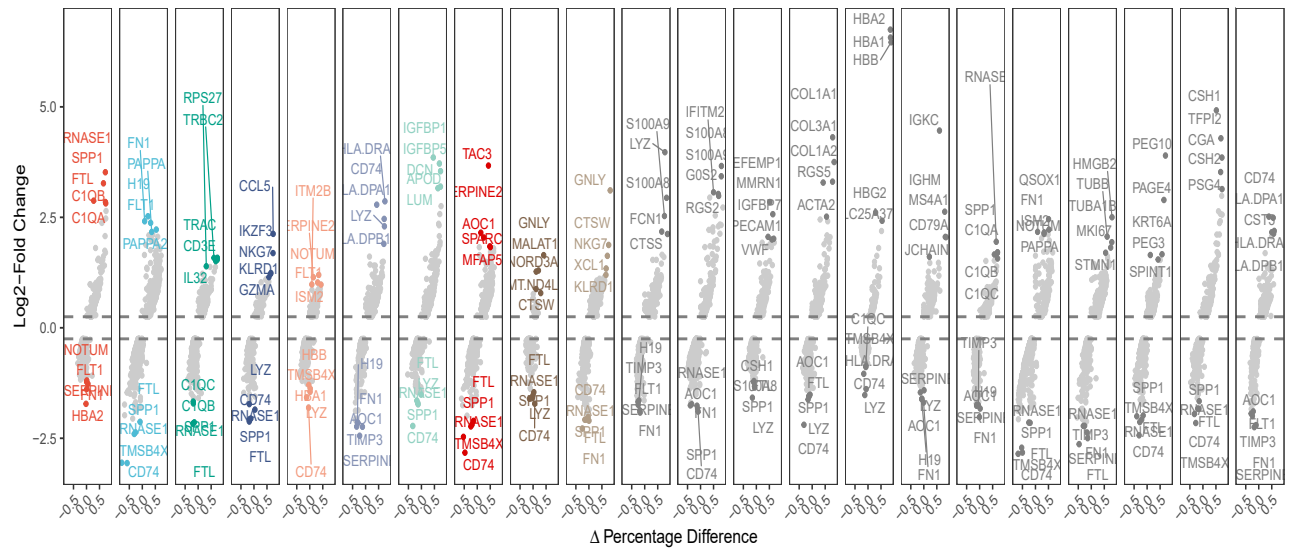

Supplement: Supplementary Figure 1 — Heatmap (A) and Volcano plot (B) show makers of each cell cluster. [file DataSheet_1.zip › supplementary FIG/FIGS1.pdf]

A

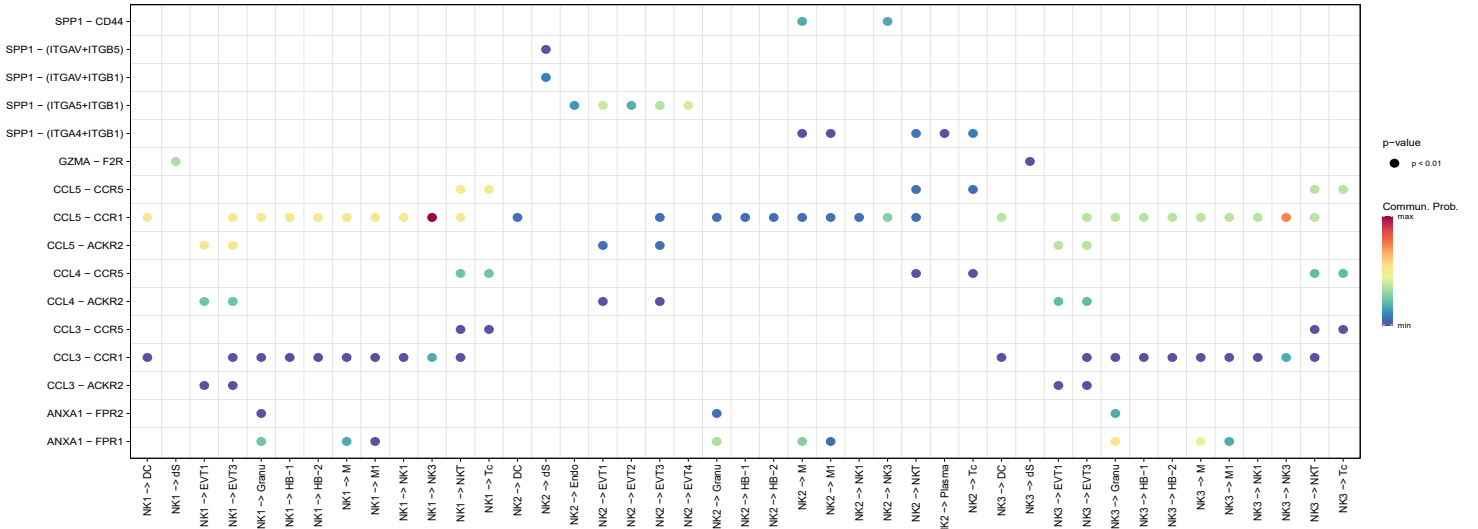

B

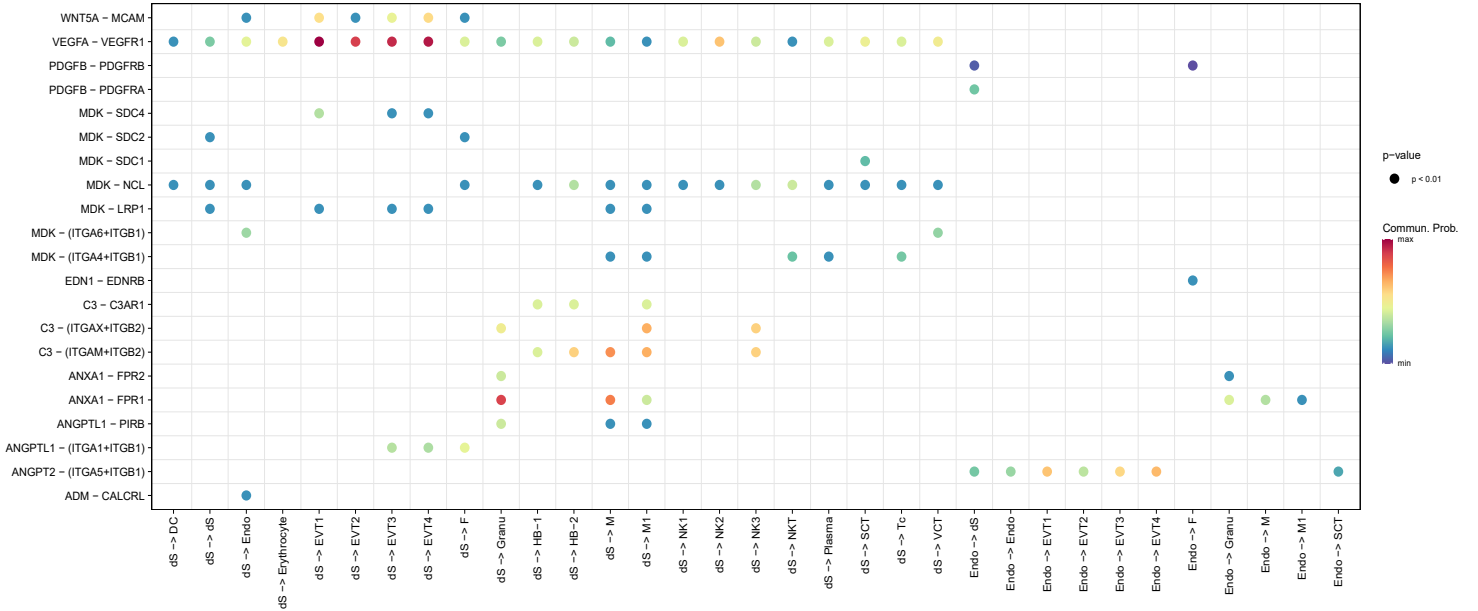

Supplement: Supplementary Figure 1 — Heatmap (A) and Volcano plot (B) show makers of each cell cluster. [file DataSheet_1.zip › supplementary FIG/FIGS2.pdf]

A

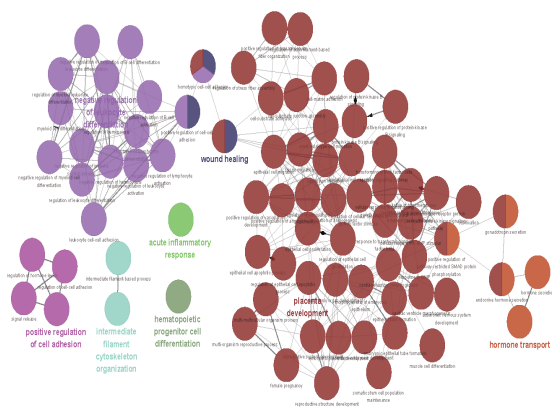

B

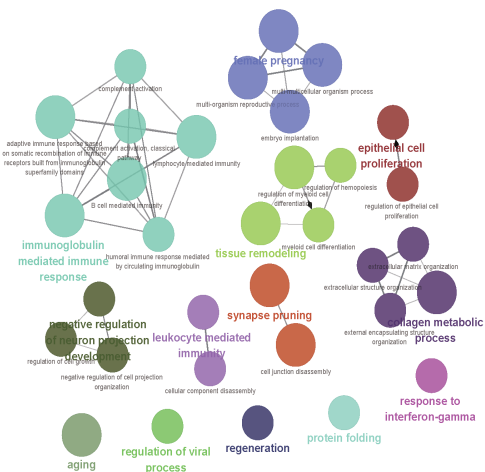

C

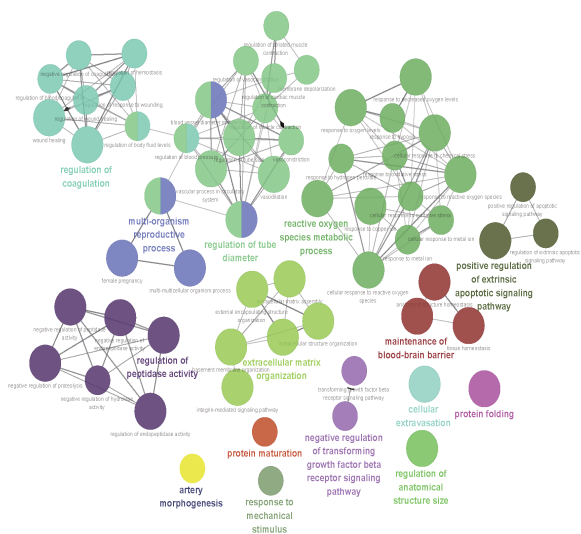

D

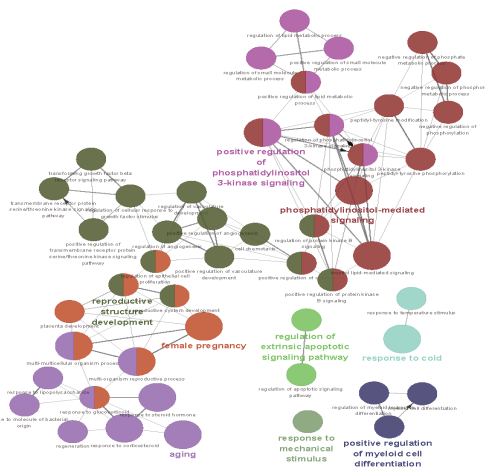

F

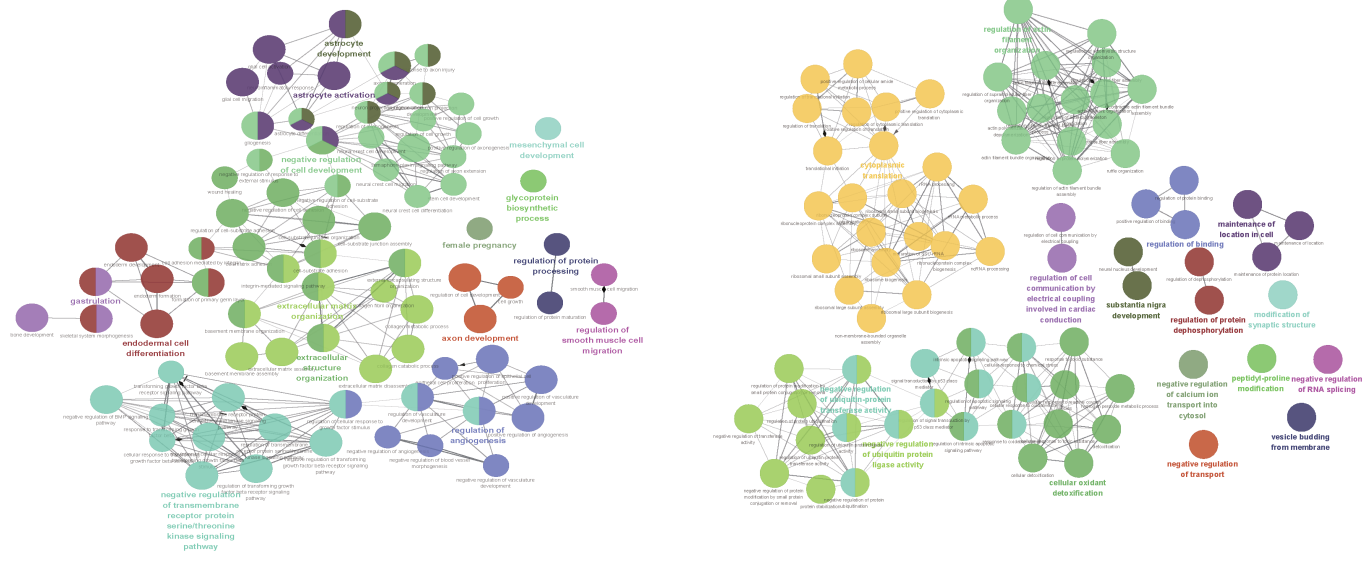

Supplement: Supplementary Figure 1 — Heatmap (A) and Volcano plot (B) show makers of each cell cluster. [file DataSheet_1.zip › supplementary FIG/FIGS3.pdf]

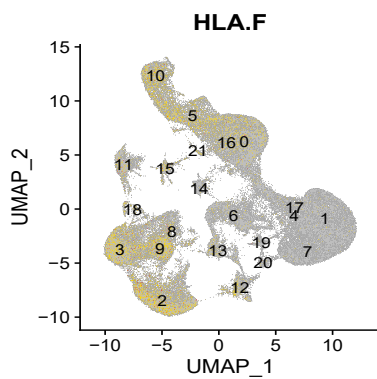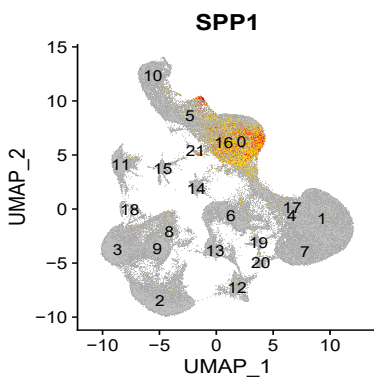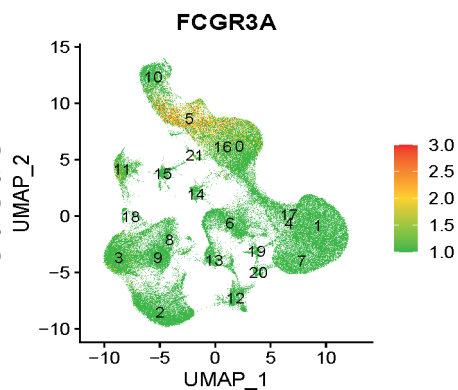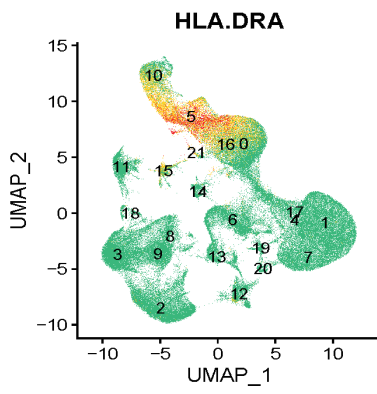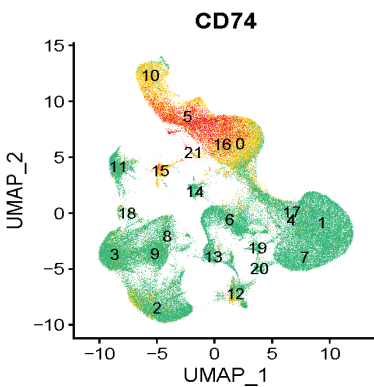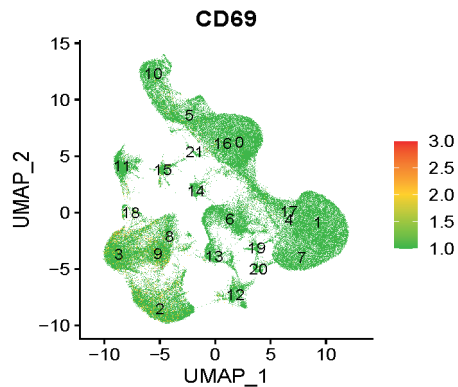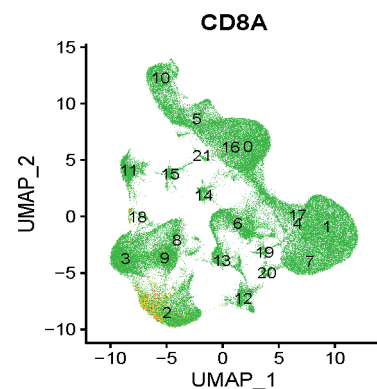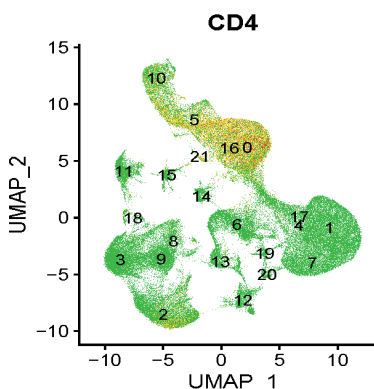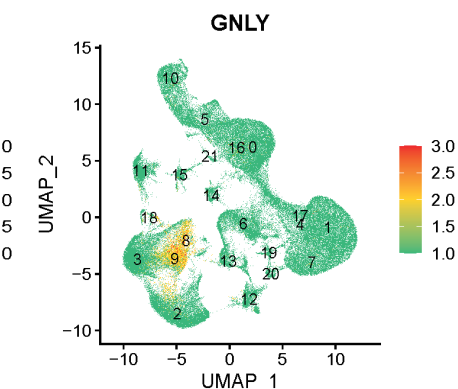

Supplement: Supplementary Figure 1 — Heatmap (A) and Volcano plot (B) show makers of each cell cluster. [file DataSheet_1.zip › supplementary FIG/FIGS4 HLA-F distribution.pdf]

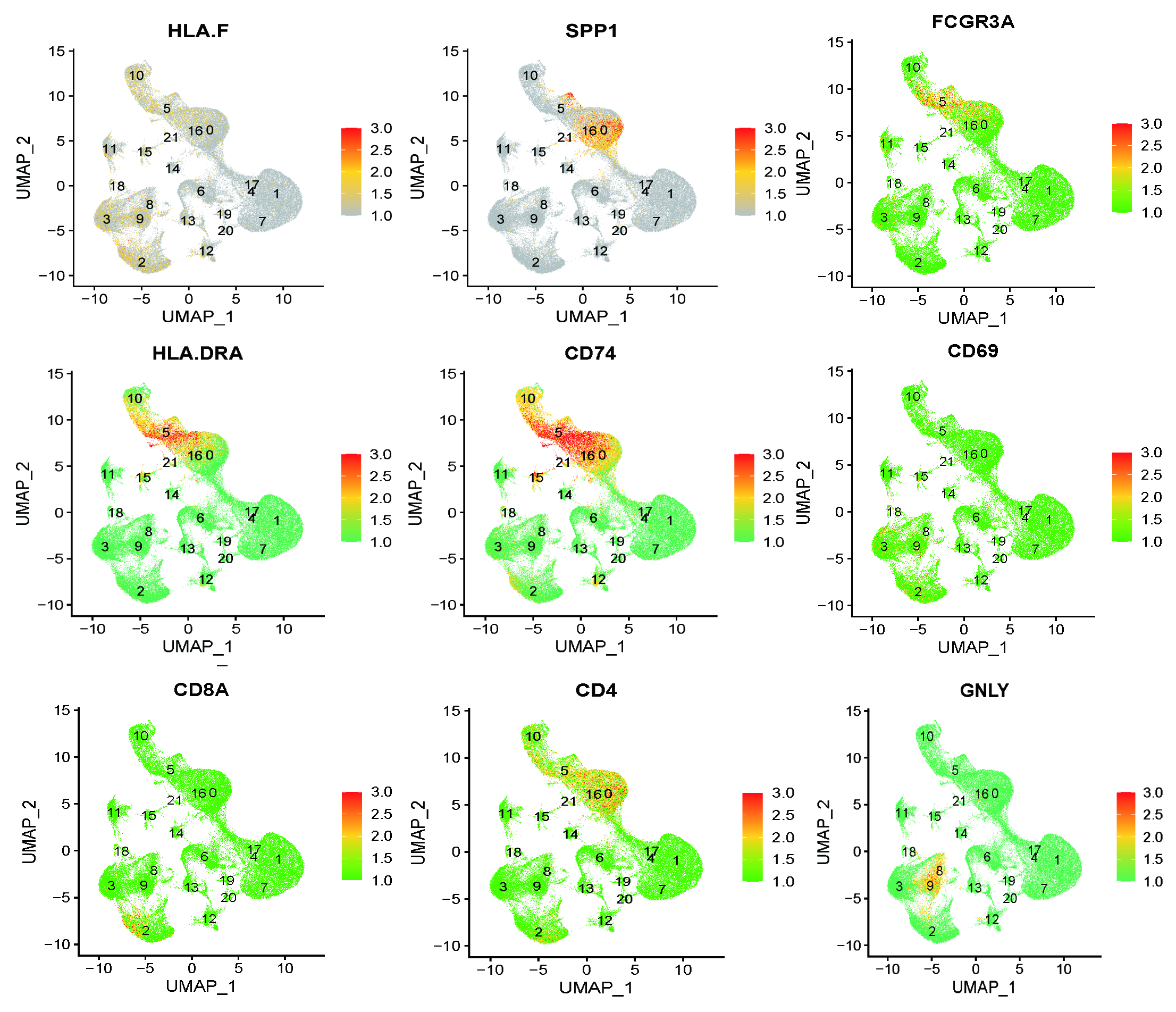

Supplement: Supplementary Figure 1 — Heatmap (A) and Volcano plot (B) show makers of each cell cluster. [file DataSheet_1.zip › supplementary FIG/FIGS4 HLA-F distribution.tif]
